# Comparative Metabolomics Reveals Key Determinants in the Flavor and Nutritional Value of Coconut by HS-SPME/GC-MS and UHPLC-MS/MS

**DOI:** 10.3390/metabo12080691

**Published:** 2022-07-26

**Authors:** Hao Guo, Jun Lai, Chun Li, Haihong Zhou, Chao Wang, Weizhen Ye, Yue Zhong, Xuecheng Zhao, Feng Zhang, Jun Yang, Shouchuang Wang

**Affiliations:** 1 College of Tropical Crops, Hainan University, Haikou 570228, China; haoguo@hainanu.edu.cn (H.G.); jun.lai@hainanu.edu.cn (J.L.); chun.li@hainanu.edu.cn (C.L.); haihong.zhou@hainanu.edu.cn (H.Z.); chaowang@hainanu.edu.cn (C.W.); weizhen.ye@hainanu.edu.cn (W.Y.); yue.zhong@hainanu.edu.cn (Y.Z.); 2 Hainan Yazhou Bay Seed Laboratory, Sanya Nanfan Research Institute of Hainan University, Sanya 572025, China; xczhao@hainanu.edu.cn; 3 National Key Laboratory of Crop Genetic Improvement and National Center of Plant Gene Research (Wuhan), Huazhong Agricultural University, Wuhan 430070, China; zhangfeng@mail.hzau.edu.cn

**Keywords:** coconut, flavor, nutritional value, HS-SPME/GC-MS, UHPLC-MS/MS

## Abstract

Coconut is a tropical fruit whose flesh has high flavor quality and nutritional value; however, the differences between coconut varieties are still unclear. Here, volatiles and non-volatiles were profiled at three ripening stages by HS-SPME/GC-MS and UHPLC-MS/MS in two coconut varieties (Hainan Tall, HT and Green Dwarf, GD). Four metabolite classes of volatiles were associated with good aroma including hydrocarbons, benzenoids, alcohols and esters, and these volatiles were generally higher in GD, especially at 7 and 9 months of coconut growth. Pathway-based metabolomics revealed that flavonols and their derivatives were significantly enriched in HT, and some of these metabolites were key determinants of HT flesh bitterness, including kaempferol 7-*O*-glucoside, a known bitter metabolite. Despite the overall accumulation of amino acids, including L-alanine, L-serine and L-methionine in GD, comparative metabolomics revealed that HT flesh provides a higher content of vitamins than GD. This study sheds light on the metabolic pathways and key metabolites differentiating the flesh flavor quality and nutritional value among coconut varieties, and reveals the possible mechanisms of flavor formation and regulation in coconut fruits.

## 1. Introduction

Flavor and nutritional value are the main factors in the fruit selection of humans. The flavor of any food is a combination of interactions between olfaction and taste [1]. Many volatile compounds can activate olfactory receptors and become the main factor for consumer preference, so they are essential for good flavor [2]. Previous studies have shown that alcohols, esters, aldehydes and ketones are the main fruit volatiles [3,4,5]. While most esters have a fragrance associated with fruit and flowers, aldehydes and ketones, formed by alcohol oxidation, have fruity and creamy aromas [6,7]. Specialized metabolites can also affect the taste diversity of fruits and vegetables [8]. For example, sugars and acids activate taste receptors, and flavor can be attained by increasing sugar and acid contents in many fruits [9,10,11,12]. Flavonoids, specifically flavonol glycosides, are the critical factors causing bitterness and astringency in some plants [11,12,13,14]. Despite flavor playing a critical role in consumer fruit selection, obtaining dietary nutrition is the key to human health, and fruits make a substantial nutritional contribution to the human diet [15]. Fruits are rich in amino acids and vitamins which are essential nutrients to maintain life [16,17,18]. Passion fruit seeds contained the 17 amino acids that are found naturally in plant protein [16]. Strawberries are rich in water-soluble vitamin C [18]. However, accumulation of these nutritional compounds can vary greatly between different varieties of the same species [19,20]. Amino acid levels fluctuate greatly in rice seed populations, and some amino acids could not be detected in a few varieties [21]. There are also significant differences in the content of vitamins such as nicotinamide among different varieties of rice and tomato [22,23]. In addition, the content of metabolites varies between different growth stages of the same fruit; therefore, selecting a fruit variety at the specific development stage that provides flavor and nutritional value remains a challenge [24,25].

In recent years, metabolomics has progressed in many fields, and metabolomics is now used widely to evaluate the flavor and nutritional quality of food [26,27]. The main techniques for analyzing volatiles, including gas chromatography–mass spectrometry (GC-MS), two-dimensional gas chromatography (GC × GC), and headspace solid-phase microextraction coupled to gas chromatography–mass spectrometry (HS-SPME/GC-MS), have been applied to determine the chemical compounds that contribute largely to the flavors of many fruits and vegetables [28,29,30]. GC-MS was used to explore the composition of the aroma of various tropical fruits, and these studies determined that myrcene, linalool and geraniol were the main components of the aroma [31,32,33,34,35]. However, GC-MS is not suitable for the detection of non-volatile metabolites, while liquid chromatography coupled with mass spectrometry (LC-MS) is widely used in the detection of such metabolites [36,37]. Among LC-MS approaches, mass spectrometers commonly utilized include the ultra-high-pressure liquid chromatography orbitrap mass spectrometer (UHPLC-Orbitrap-MS) and the ultra-high-pressure liquid chromatography triple quadrupole mass spectrometer (UHPLC-TQ-MS) [34,35]. Orbitrap-MS can obtain high-resolution data for accurate exact mass and elemental composition assignment, while TQ-MS is often used for quantitative analysis due to its high sensitivity. Several studies have demonstrated the power of using these technologies for volatile and non-volatile metabolite analysis [34,38]. For example, the nutritional components of six cruciferous vegetables were investigated by HS-SPME/GC-MS non-targeted analysis, and a total of 55 nutritional metabolites and 190 volatiles were detected [38]. In a separate study, a total of 47 nutritional components in the flavedo, albedo and pulp of pummelo fruits were determined by UHPLC-TQ-MS targeted analysis. Advancements in the field have made metabolomics an effective tool to explore flavor and nutritional value.

Coconut (*Cocos nucifera* L.) is an important economic crop, mainly distributed in tropical regions such as Indonesia, Philippines, India, and Brazil [5]. In some tropical countries and regions, millions of people consume food products containing coconut daily, especially coconut flesh. Coconut flesh is also popular around the world as a snack and powder because of its distinctive flavor and high nutritional value [39]. Coconut can be divided into two distinct varieties: Cocos nucifera tall (*Cn. tall*) and Cocos nucifera dwarf (*Cn. dwarf*) [40]. *Cn. tall* has a strong tolerance to abiotic stresses, but it produces less fruit and the fruit have poor taste [40,41]. In contrast, the flesh of *Cn. dwarf* is loved by consumers [42]. However, the growth stage that provides the best flavor and nutritional quality of coconut has yet to be identified, and the molecular mechanisms controlling the differences in flavor and nutrition between the two coconut varieties remain to be investigated. 

Here, we used HS-SPME/GC-MS and UHPLC-MS/MS to explore the differences in flavor and nutrition of ripe coconut flesh between three growth stages (Figure S1). Two representative coconut varieties, a *Cn. tall* named Hainan Tall (HT) and a *Cn. dwarf* coconut named Green Dwarf (GD), were used for metabolomic comparison. We found that GD has a higher abundance of flavorful volatiles, among which hydrocarbons, benzenoids, alcohols and esters were higher overall in GD. We determined that high contents of flavonol and their derivatives are the main cause of the bitterness of HT using pathway-based metabolomics analysis. Metabolites associated with nutritional value also differed between the two coconut varieties. Vitamin B3 and its precursor metabolites were highly accumulated in HT, but methyl nicotinate, a downstream metabolite of B3, was significantly higher in GD than in HT. Most amino acids also showed significant enrichment in GD, such as L-alanine, L-serine and L-methionine. This study analyzed the differences in flavor and nutrition of two coconut varieties, and determined the differences in metabolite accumulation patterns. Thus, we created a valuable resource that could be utilized for the improvement of coconut flavor through crop breeding or genetic engineering. 

## 2. Results

### 2.1. Identification and Analysis of Coconut Varieties Volatile Content

**Figure 1:**
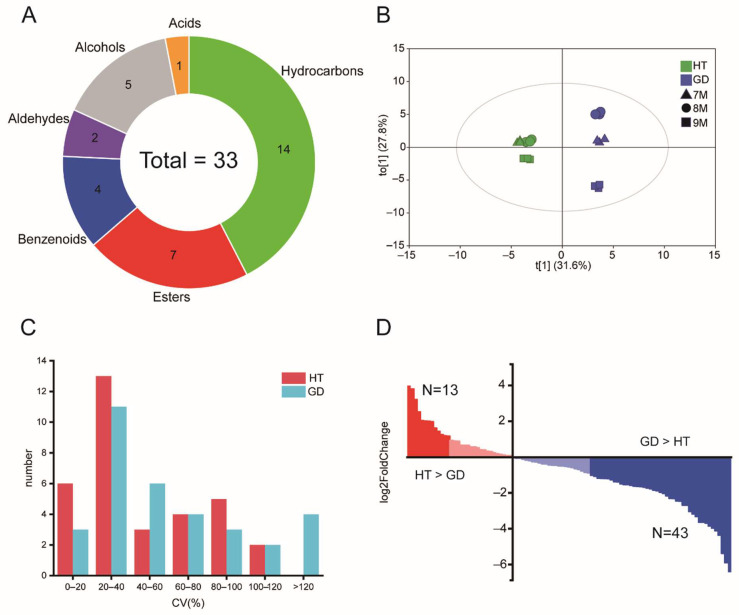
Statistical analysis of volatiles. (**A**) Metabolite classes of the volatiles detected in HS-SPME/GCMS analysis. (**B**) OPLS-DA scores plot analysis of detected volatiles between different varieties of coconut over three growth stages. (**C**) Coefficient of variation (CV) analysis redundant of the metabolome during flesh growth of HT and GD. (**D**) The distribution of the fold change between HT and GD. The metabolites are ordered based on their fold change between HT and GD.

Fruit aroma is a key determinant that affects fruit flavor, customer selection and competitiveness in the market. To investigate the aroma volatiles of coconut flesh, two varieties with contrasting flavor qualities, HT and GD, were utilized to detect organic volatiles in the flesh. Three growth stages, from 7 months of growth (termed 7M in this study) to 9 months (9M), were used to explore the differences in volatile content. A total of 33 volatiles were detected using untargeted HS-SPME/GC-MS in the flesh of the two coconut varieties (Tables S1 and S2). These volatiles were composed of hydrocarbons, esters, benzenoids, aldehydes, alcohols and acids (Figure 1A). The number of hydrocarbons detected was the largest (reaching 14), while just one acid could be detected. Thus, multiple metabolite classes important for fruit aroma were measured in this analysis.

To determine the differences in total volatile composition during coconut flesh growth, orthogonal partial least squares-discriminant analysis (OPLS-DA) was performed on the relative abundance of volatiles (Figure 1B). Then, we verified the model through the permutation test and the values of R2Y and Q2 are 0.985 and 0.968, respectively. This shows that the fitting accuracy of the model is very high. The analysis determined that three biological replicates of samples from different growth stages were clustered together clearly, indicating that the samples in this study were consistent. The volatile profiles from coconut fruit at 7M and 8M were clustered together and separated from 9M in HT. This result indicates that the difference between 7M and 8M was not significant, but the volatile content at 9M differed from the other two growth stages. There were notable differences in the relative content of each month in GD. The coefficient of variation (CV) statistic showed that the CV of 20–40% is the most in HT and GD, with 13 and 11, respectively, of which only GD has a CV greater than 120% (Figure 1C). These data indicate that GD undergoes dynamic changes in volatile content between different stages. To analyze the dynamic variations of the volatiles between HT and GD during flesh growth, differentially accumulated metabolites (DAMs) analysis was used (Table S3). The total number of volatiles significantly accumulated in HT was 13, while GD significantly accumulated 43 volatiles (Figure 1D). These results suggest that the content of volatiles in GD varies more between different growth stages, and the content of volatiles in GD is higher than the volatile content in HT.

### 2.2. Dynamic Changes of Volatiles during Flesh Growth in HT and GD

**Figure 2:**
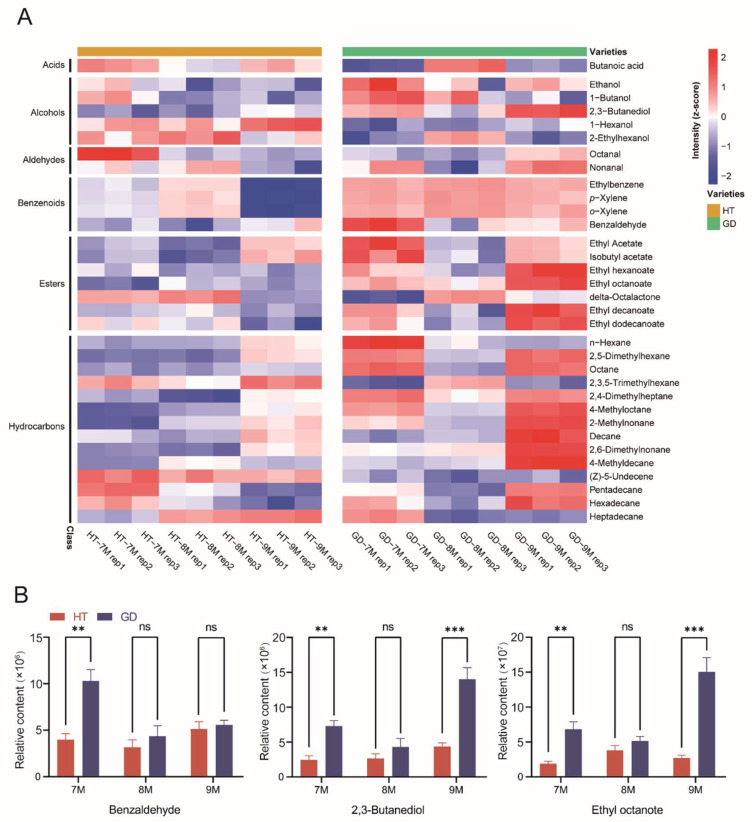
Analysis of coconut flesh volatile accumulation patterns between two coconut varieties over three growth stages. (**A**) The levels of volatile of coconut flesh in two coconut varieties over three growth stages are shown in the heatmaps. Volatiles were arranged by metabolite classes. (**B**) Comparative analysis of volatiles in the flesh of two coconut varieties at three different growth stages. Student’s *t* test was performed (ns: not significant, ** *p* < 0.01, *** *p* < 0.001).

To better understand the accumulation patterns of different metabolite classes of volatiles during ripening between two coconut varieties, specific compounds, identified in the multivariate statistical analysis, were analyzed according to the heatmap (Figure 2A). The heatmap indicated that four classes of volatiles—alcohols, benzenoids, esters and hydrocarbons—had higher content in GD than HT from 7M to 9M. Since these four metabolite classes of volatiles, which produce the fragrances of fruit, flowers and cream. We concluded that they were responsible for the pleasant aroma of GD. Additionally, the key factors leading to the difference in flavor were analyzed between GD and HT. The content of benzaldehyde with cherry and nutty aromas was significantly higher in GD (Figure 2B). The levels of 2,3-butanediol and ethyl octanoate, the most representative of floral flavor volatiles, were also enriched in GD when compared to HT. However, the content of these volatiles was the lowest at 8M in GD, and there were no significant differences between the two varieties for most of the detected volatiles at this growth stage. Some other key volatiles such as ethyl decanoate and ethyl dodecanoate with grape and leaf aromas showed the same trend [43] (Figure S2). These results suggest that, at the 7M and 9M growth stages, the coconut flesh of GD had a more consumer-friendly volatile flavor than HT, but this difference was not remarkable at 8M.

### 2.3. Identification of Metabolite Signals by UHPLC-Orbitrap-MS

**Figure 3:**
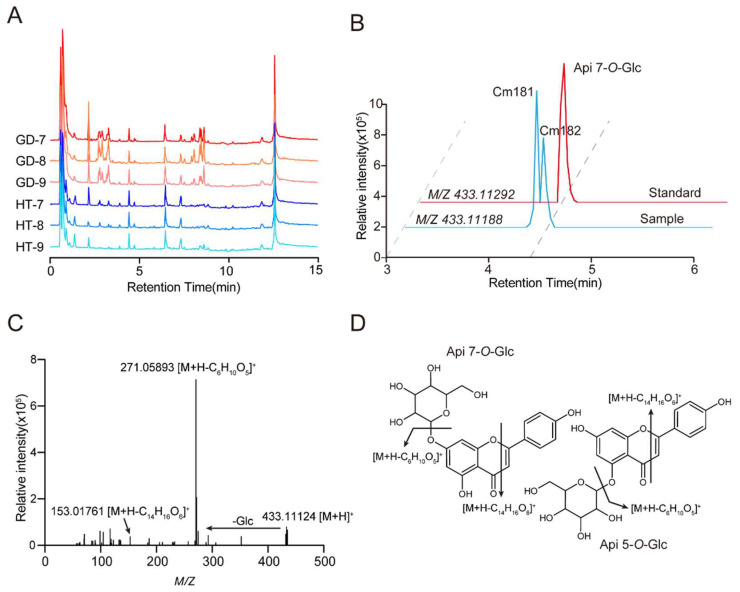
Detection and identification of secondary metabolites by UHPLC–HRMS. (**A**) TIC (total ion chromatogram) of metabolites in different varieties and stages of coconut flesh obtained from UHPLC–MS/MS analysis. (**B**) EIC (extracted ion chromatogram) of apigenin 5-*O*-glucoside and apigenin 7-*O*-glucoside detected at 4.28 min and 4.35 min in sample. Apigenin 7-*O*-glucoside detected at 4.35 min in standard. (**C**) MS/MS spectra at *m*/*z* 433.11124, and the metabolite was identified as apigenin 7-*O*-glucoside. (**D**) The molecular structure of the apigenin 7-*O*-glucoside and apigenin 5-*O*-glucoside and their general fragmentation rules.

Fruits are rich in primary and secondary metabolites, which determine taste and nutritional value. To dissect the differences in taste and nutritional value among these two coconut varieties, metabolic profiling via UHPLC-MS/MS was performed based on a nontargeted method combined with a targeted metabolic profiling method. In short, we conducted a LC–MS-based nontargeted metabolomics analysis in which 4409 mass spectrometric signals were detected in mixed samples after filtering by signal-to-noise ratio and subtract background (Figure 3A). Additionally, we found that 3864 metabolic signals had secondary mass spectra. Next, we annotated the metabolites based on the accurate precursor ions (Q1), retention times (RT), mass spectral fragmentation information, the standard and local databases.

In these samples, metabolic signals (Cm181 and Cm182) with retention time of 4.28 min and 4.35 min were detected, and with an exact *m*/*z* value of 433.11188 for their precursor ion (Figure 3B). Moreover, similar characteristic fragments of 433.11124, 271.05893 and 153.01761. And the ion 153.01761, was observed in the MS fragment, which was due to the characteristic fragment of flavonoids. It’s speculated that the mass spectrum signals are apigenin *O*-glucoside (Figure 3C,D). Through comparison with the standard, the metabolic signal Cm182 was identified as apigenin 7-*O*-glucoside due to the same mass spectral fragmentation information and retention time (Figure 3B). Therefore, we speculate that Cm181 may be the apigenin 5-*O*-glucoside, because of the same fragment information and earlier retention time. A total of 237 metabolites were identified by UHPLC-HRMS (Table S4). These metabolites included amino acids and derivatives (85), organic acids and derivatives (47), flavonoids (40), lipids (29), sugars (22) and vitamins (14).

### 2.4. Metabolic Profiles by UHPLC-TQ-MS of Two Coconuts Varieties

**Figure 4:**
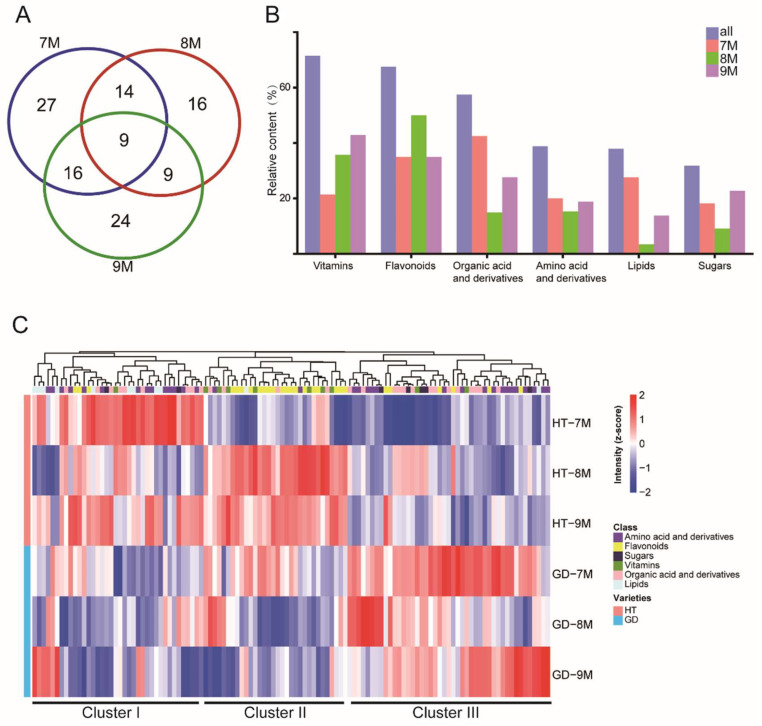
Differential accumulation metabolite analysis. (**A**) Venn diagram shows the number of differentially accumulated metabolites among three growth stages. (**B**) Statistical analysis of differentially accumulated metabolites in different metabolite classes. (**C**) The levels of nonvolatile of coconut flesh in two coconut varieties over three growth stages are shown the heatmaps. Hierarchical cluster analysis of differentially accumulated metabolites. Each column in the figure represents a metabolite, each row represents either GD or HT coconut varieties at three different growth stages.

Next, the 237 metabolites were quantified by UHPLC-TQ-MS (Table S5). We used principal component analysis (PCA) to differentiate the metabolomes of these coconut varieties at different growth stages (Figure S3). To analyze the individual metabolite features contributing to the differences in the model, DAMs were calculated between HT and GD during coconut ripening (Figure 4A). A total of 115 metabolites differed significantly in at least one stage; the number of DAMs was largest in the 7M stage and was the least at 8M (Table S6). There were nine metabolites that showed distinct differences in all three stages, including five flavonoids, two amino acids and derivatives, and two organic acids and derivatives. Furthermore, to dissect which categories of metabolites were most different, we further analyzed these DAMs by metabolite class. Figure 4B showed that vitamins, followed by flavonoids, accounted for most DAMs. The number of DAMs that were vitamins was the least at the 7M stage, and the number of differentially accumulated flavonoids was the largest at the 8M stage. However, less than 50% of amino acid and their derivatives were significantly different between the two varieties. These results suggested that vitamins were the main metabolite class differentiating the two coconut varieties. 

Next, the accumulation pattern of 115 DAMs at the three growth stages was visualized by heatmap (Figure 4C). Overall, these metabolites showed some accumulation patterns, including amino acids, amino acid derivatives and flavonoids. Most amino acids and amino acid derivatives were located in cluster III. Amino acid and amino acid derivative abundances were low in HT at all three growth stages, while they accumulated in GD. Most flavonoids were located in cluster II, and they showed a higher accumulation at the 8M and 9M growth stages of HT. These results suggest that metabolites such as amino acids and flavonoids differ globally between the two coconut varieties, and changes in the biosynthetic pathway could be responsible for these differences.

### 2.5. Flavor Biosynthesis during Ripening of Different Coconut Varieties

Since sugars and organic acids are the key determinants of taste, we sought to compare these metabolites between the two coconut varieties. There was no significant difference in the relative content of sugars between GD and HT, including sugars with a pronounced sweet taste such as DL-arabinose, D-(+)-glucose, D-(+)-mannose, D-(+)-galactose and D-(+)-sucrose (Figure S4). However, the content of a few organic acids in GD was significantly higher than that in HT, including citric acid and succinic acid, and these metabolites could enrich the taste of the fruit (Figure S5). While these results show that there is no significant difference in the metabolites responsible for fruit sweetness between the two coconuts’ flesh, the increase in organic acids could create a better coconut flavor, and account for the popularity of GD over HT.

**Figure 5:**
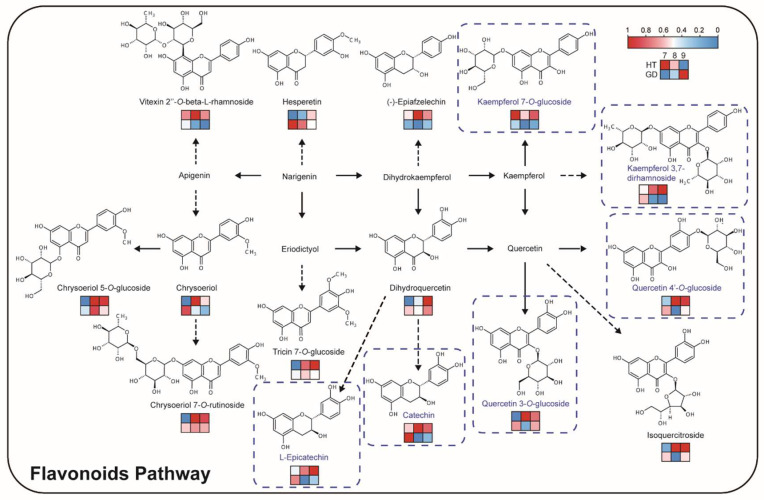
Flavonoid biosynthesis pathway in the coconut flesh. The heatmaps are drawn according to the relative content of metabolites, and the contents of each metabolite are normalized. Columns and rows in the heatmap represent stages and varieties, respectively. In each heatmap, the upper row represents HT, the lower row represents GD. In the columns of each heatmap, 7M, 8M and 9M are represented from left to right. The metabolites with blue frames have a bitter taste.

Flavonoids are most abundant in HT, so the flavonoid biosynthesis pathway may account for the bitter taste of HT flesh (Figure 4C). A pathway including 15 flavonoids was constructed, including flavonols, flavonoids, flavanones and catechins (Figure 5). Seven glycosylated flavonols accumulated in HT at multiple growth stages. One of these glycosylated flavonols, kaempferol 7-*O*-glucoside, that was determined to be the key bitter and astringent tasting compound in the tea plant, was over four times more abundant at the 9M growth stage compared to the 7M growth stage in HT. Other precursors and products of flavonoids displayed the same accumulation pattern, including (−)-epiafzelechin and vitexin 2″-*O*-beta-L-rhamnoside. Among them, catechin and L-epicatechin are both bitter and astringent compounds, and accumulate in HT compared to GD. These flavonoid accumulation patterns indicate that the total accumulation of flavonols and their derivatives may be the main reason for the poor taste of HT.

### 2.6. Evaluation of Nutritional Value of Different Coconut Varieties

**Figure 6:**
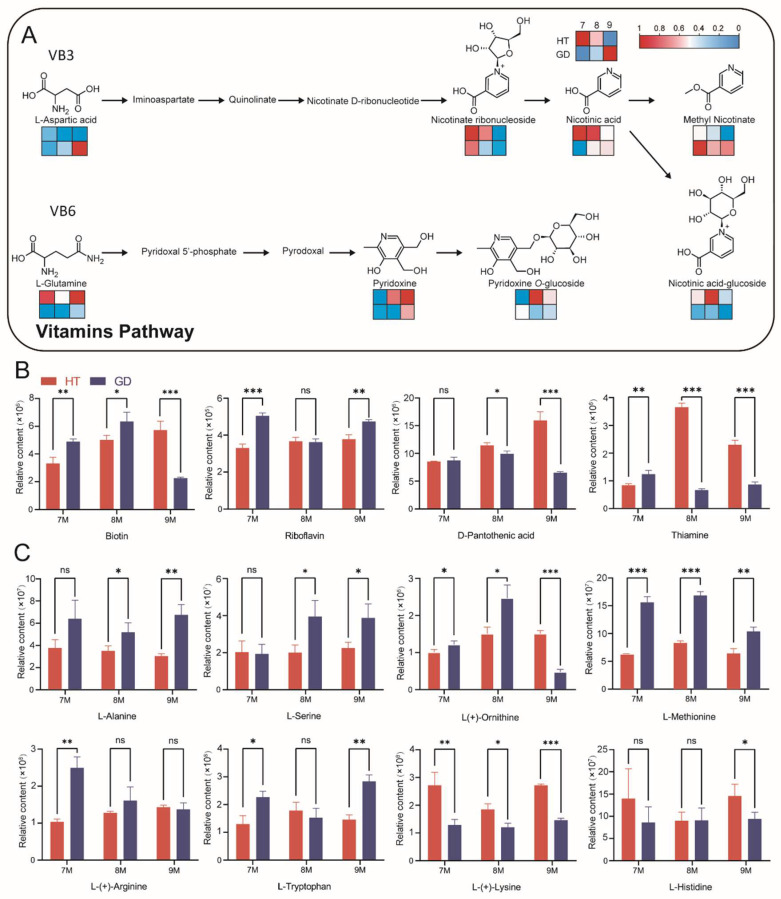
Comparative analysis of nutritional metabolites in the flesh of two coconut varieties across three growth stages. (**A**) Vitamin pathways in coconut flesh. The heatmaps are drawn according to the relative content of metabolites, and the contents of each metabolite were normalized. Columns and rows in the heatmap represent stages and varieties, respectively. In each heatmap, the upper row represents HT, the lower row represents GD. In the columns of each heatmap, 7M, 8M and 9M are represented from left to right. (**B**) Differences in vitamin levels between two coconut varieties across three growth stages. (**C**) Differences in amino acid levels between two coconut varieties across three growth stages. Student’s *t* test was performed (ns: not significant, * *p* < 0.05, ** *p* < 0.01, *** *p* < 0.001).

After analyzing the volatile smell and taste of the two coconuts, we focused on the comparison of metabolites significant in dietary nutrition. Vitamins and amino acids are important nutrients needed by humans. The content of vitamins in HT is higher than that in GD, and there are distinct differences in multiple metabolites in the vitamin B3 and B6 pathways (Figure 4C). Some vitamins, including nicotinic acid and pyridoxine, are more abundant in HT overall (Figure 6A). Other key vitamins also have conspicuous differences between the two coconuts, including biotin, thiamine, riboflavin and D-pantothenic acid (Figure 6B). Unlike vitamins, most amino acids accumulated notably during the growth of GD. L-methionine, L-(+)-arginine and L-tryptophan were highly abundant in GD flesh at the 7M growth stage. There were no remarkable differences in L-alanine and L-serine content between GD and HT at the 7M growth stage; however, these metabolites were significantly higher in GD at 8M and 9M (Figure 6C). In contrast, L-(+)-lysine accumulation was higher in HT than it was in GD at every growth stage. From these results, we concluded that the nutrient contents of the two coconuts are diverse, and while the accumulation of amino acids overall was relatively abundant in GD, the vitamin content is higher in HT.

## 3. Conclusions

Flavor and nutrition are essential elements for the consumer. In this study, we explored differences in flavor and nutrition of mature coconut flesh among three key stages, attempted to unravel the metabolic mechanisms regarding the biosynthesis of flavor and nutrition compounds in coconut, and the primary and secondary metabolites of coconut flesh were comprehensively analyzed, in which more than 200 compounds were reported for the first time. Our results revealed that the main components of the coconut flesh included volatile substances and non-volatile substances. Some of these volatile odorous esters, such as caprylic acid ethyl ester, may con-tribute to the more fragrant GD, while some glycosylated flavonols, such as quercetin-4′-*O*-glucoside, may cause the HT to have a poorer taste; regarding vitamin amino acids analysis, there are more nutrients in HT. Therefore, compared with previous studies, our results have the advantage of comprehensively clarifying the chemical composition of coconut flesh.

## 4. Discussion

Metabolites are not only the basis of plant survival, but are also the source of flavor and nutrition [44,45]. Plant metabolomics has developed rapidly in the past decade, especially GC-MS and LC-MS, for utilization in the detection of key flavor and nutritional compounds in fruits and vegetables [46,47]. For example, Tieman et al. used GC-MS to identify the chemicals in modern, heirloom and wild tomatoes that contributed the most to flavor and consumer liking [2]. The compounds that impact the consumer preference of strawberry preserves were also investigated by using untargeted LC-MS flavoromics analysis [48]. Thus, metabolomics is an effective and meaningful tool to measure the flavor and nutrition of food. Indeed, a total of 217 metabolites have been identified from coconut water, including 54 organic acids, 44 amino acids, 30 sugars and other metabolites based on LC-MS. Moreover, 25 compounds were identified based on GC-IMS, including 12 esters, 5 alcohols, 3 ketones and other volatiles. The same E-tongue can also be used to evaluate umami, sweetness and bitterness [49]. In this study, we detected 33 volatiles and 237 compounds among three stages of coconut flesh development in two coconut varieties using HS-SPME/GC-MS and UHPLC-MS, respectively. This suggested that although coconut water is widely consumed, the coconut flesh is richer in metabolite diversity. Among the different growth stages, the metabolome underwent dramatic dynamic changes that are comparable to other fruits, and flavonoids and vitamins accounted for the most significant differences in this study [50,51,52].

Comparing coconut varieties with contrasting traits allowed us to study biosynthetic pathway differences, and key compounds which influence flavor in other fruit bearing species were significant in our results. For example, ethyl acetate, ethyl caproate and ethyl caprylate have been reported to have obvious fruity and floral aromas [53,54,55], and the content of these volatiles is significantly higher in GD than HT. This difference may account for GD being more fragrant than HT. However, not all fragrance compounds are increased in GD; for example, 2-ethyl-1-hexanol has been reported to have a rose aroma, and the content in HT is higher than GD [56]. We speculate that these volatiles may give HT its distinctive smell. In addition to volatiles, there are also large differences in some compounds that affect taste. As shown in Figure 4 and Figure 5, the level of flavonoids was relatively higher in HT compared to GD varieties. There are many key enzymes in the two pathways, such as cinnamic acid hydroxylase (*C4H*), 4 coumarate-CoA ligase (*4CL*), flavonoid 3-hydroxylase (*F3H*) and trehalose phosphate synthase (*TPS*) [57,58]. Multi-omics analysis shows that *C4H* was affected by DNA methylation, causing flavonoids metabolism diversity and revealing the mechanism underlying the edaphic adaptation in wild barley [59]. Natural variation of *4CL* was found to lead biosynthesis of coumaric acid derivatives [60], and overexpressed F3H can increase dihydrokaempferol content in tobacco [61]. *TPS* catalysis can produce trehalose-6-phosphate (*T6P*), which can adjust the sugar level [62]. Thus, we surmise that the variety in flavonoids are caused by the differential transcription levels of these key enzymes in HT and GD varieties, while *TPS* has no differential expression in different varieties. In addition, sugar is the main source of sweetness in fruit, and fruit with a moderately higher content of sugar was favored by consumers [63,64]. However, the content of sugars, including sucrose, glucose and mannose, was not significantly different between the two coconuts in the three stages (Figure S4). Thus, we believe that the sweetness of the flesh is not the reason for the difference in flavor between the two varieties.

Nutrient quantity obtained from fruits and vegetables has been a concern all over the world because of malnutrition in humans. Studies have shown differences in nutrients at different stages of plant development; for example, carotenoids, such as violaxanthin, neoxanthin, together with vitamin K1, were higher in the seedlings of *Brassica rapa subsp. chinensis var. parachinensis* [65,66]. In this study, we detected classes of compounds that were beneficial to human health, including primary and secondary metabolites. Some amino acids such as L-theanine, L-valine and L-glutamic acid were accumulated at high abundances at 7M and 9M in GD (Table S5). This result indicates that humans can obtain higher levels of amino acids by consuming coconut flesh at 7M and 9M stages. Secondary metabolites such as vitamins, flavonoids and organic acids can promote digestion and enhance immunity [67], and they have different accumulation patterns in different varieties of the same fruit [68]. This study detected 14 vitamins, 40 flavonoids and 47 organic acids among the two varieties of coconut. Although HT has a worse flavor, it is higher than GD in some nutritional values such as vitamin B1, vitamin B3, vitamin B5 and vitamin B6. Flavonoids that provide a bitter taste of HT can prevent chronic diseases, and plants rich in flavonoids can enhance tolerance to biological or abiotic stresses [69]. Except for the high accumulation of citric acid in GD, most organic acids were not significantly different between the two varieties. These results indicate that eating HT flesh can provide more vitamins, while eating GD flesh can provide a higher content of organic acids, amino acids and amino acid derivatives.

This research accomplished the metabolomics characterization and nutritional analysis of coconut flesh by using GC-MS and LC-MS based on the metabolomics approaches. We performed metabolite profiling on the major flavor and nutritional pathways (flavonoid metabolism and vitamin metabolism) to investigate metabolic flux through the pathways, and the relationships between flavor precursors and products in each pathway. Based on these results, possible metabolic mechanisms of key flavor and nutrition formation and regulation in coconut were identified and discussed.

## 5. Materials and Methods

### 5.1. Plant Materials and Sampling

To investigate the coconut metabolome, coconut flesh of different varieties was analyzed. Coconuts were collected from trees maintained at the Coconut Research Institute (Wenchang, China). We harvested fruit at different stages for analysis, and the selection of materials was obtained from two representative coconut varieties, HT and GD. Varieties were harvested randomly from trees in three positions and pooled for each biological replicate. After the fruit was picked, flesh samples were removed from the shell with a spoon and chopped, and then collected in a 50 mL polypropylene centrifuge tube which was placed in liquid nitrogen.

### 5.2. Reagents and Standards

HPLC-grade acetonitrile, acetic acid and methanol were purchased from Merck (Darmstadt, Germany); water was purified with a MilliQ ULTRA purification system (Millipore, Vimodrone, Italy). The internal standard used in this study was lidocaine, which was bought from Shanghai New Asiatic Pharmaceuticals Co., Ltd. (http://www.xinyapharm.com/ accessed on 10 August 2021). N-alkanes series (C6–C40) (purity > 98%) were purchased from Sigma Aldrich (St. Louis, MO, USA).

### 5.3. Sample Preparation and Extraction

The fresh tissues of different varieties were ground using a mixer mill (MM 400, Retsch) with a zirconia bead for 30 s at 30 Hz.

Then, 1 g powder was weighed and transferred into a 22-mL glass headspace vial, incubated for 10 min at 37 °C, and then 2.2 g of CaCl_2_·2H_2_O and 1 mL of a 100 mM EDTA-NaOH (pH 7.5) solution were added. The sample was gently mixed and sonicated for 5 min. Samples were preheated for 10 min at 50 °C and extracted for 20 min at 50 °C before GC-MS analysis [70].

Next, 100 mg powder was weighed and extracted overnight at 4 °C with 1.0 mL 70% aqueous methanol for metabolites. Following centrifugation at 12,000 rpm for 10 min, all of the supernatants were pooled and filtrated (SCAA-104, 0.22 lm pore size; ANPEL, Shanghai, China). In addition, to ensure the stability and suitability consistency of MS analysis, a quality control (QC) sample was prepared by mixing equal volumes (10 μL) of all the filtered supernatants before LC-MS analysis [71]. Additionally, we also used the internal standards (0.1 mg L^−1^ lidocaine) to divide and normalize the relative signal strength of the metabolite.

### 5.4. HS-SPME/GC-MS Analysis

Volatiles were detected on a gas chromatography apparatus (7890A GC, Agilent Technologies, Santa Clara, CA, USA) coupled to an Agilent 7000D triple quadrupole mass detector. An HP-5 MS capillary column (30 m × 0.25 mm i.d. × 0.25 μm film thickness; Agilent Technologies) was used to separate volatile compounds. The initial column temperature was 40 °C, held for 3 min, and the temperature was increased by 2 °C/min to 160 °C, then the temperature was increased by 50 °C/min to a final temperature of 300 °C and held for 3 min. The injection temperature was 270 °C, and the splitless mode was used with a 0.75-mm i.d. inlet liner (Agilent Technologies). The flow rate was 1.0 mL/min of He (99.999%). The scan range was 50–650 *m*/*z* in full scan mode.

The chromatograms of volatile compounds were extracted by MassHunter Qualitative Analysis software (Agilent Technologies, Inc., Santa Clara, CA, USA). The isolated volatile compound was identified by its mass spectrometry fragment, matched with the National Institute of Standards and Technology (NIST search 2.3) and the Human Metabolome Database (HMDB), assisted by the mixture of n-alkanes series (C6–C40) to compare the experimental retention index (RI) with the values available in the NIST MS libraries. Then, the data were taken as valid according to the relevant literature retention indices (LRIs) for manual qualitative analysis with a similar index (SI) > 80. In addition, the content of the volatile compound was calculated by dividing the peak area of the isolated compound by the peak area of the internal standard.

### 5.5. UHPLC-Orbitrap-MS Analysis

UHPLC-MS/MS analysis was performed on a Thermo Fisher Q Exactive Plus mass spectrometer (Waltham, MA, USA; Thermo, Bremen, Germany) equipped with a heated electrospray ionization (HESI) ion source, using a Thermo Scientific Vanquish ultra-high-performance liquid chromatography (UHPLC) system (Thermo Fischer Scientific, Bremen, Germany). Chromatographic separation was performed on a Waters CORTECS T3 Column (2.7 μm, 2.1 mm × 100 mm) from Waters Corporation (Milford, MA, USA). The temperature of the autosampler and column was set at 10 °C and 40 °C, respectively. The flow rate in gradient mode was 0.4 mL/min. The mobile phase was acidified water (0.04% acetic aicd in water, *v*/*v*) (mobile phase A) and acidified acetonitrile (0.04% acetic acid in acetonitrile, *v*/*v*) (mobile phase B). The linear gradient of mobile phase B was 5–95% within 0–10 min, 95% within 10–11 min, 95–5% within 11–11.1 min, and 5% within 11.1–15 min (total run time: 15 min). The samples (2 uL) were injected onto the system and analyzed in the positive electrospray ionization (ESI) mode. The mass spectrometer performed full MS and ddMS2 scans. Full MS scan optimized acquisition parameters were: resolution 70,000 full width at half maximum (FWHM); Automatic Gain Control (AGC) target 3 × 10^6^; maximum injection time 100 milliseconds (IT, the maximum time allowed to obtain the set AGC target); and scan range 120–1800 *m*/*z*. DdMS2 scan optimized acquisition parameters were: resolution 35,000 FWHM; AGC target 1 × 10^5^; maximum IT 50 milliseconds; loop count 12 and MSX count1 (TopN 12); isolation window in quadrupole 3 *m*/*z*; and specific normalized collision energy (NCE) for each precursor *m*/*z* in 20, 40, 60: Dynamic exclusion auto. During acquisition, the mass spectrum was collected by the Xcalibur 4.1 software (Thermo Fisher Scientific, San Jose, CA, USA) for metabolomic analysis.

### 5.6. UHPLC-TQ-MS Analysis

For each sample, three biological replicates were independently analyzed. The UHPLC-MS/MS analysis was carried out using a 7500 triple quadrupole-linear ion trap mass spectrometer (AB Sciex, Framingham, MA, USA). The analytical conditions were as follows: HPLC column, Waters ACQUITY UPLC HSS T3 C18 (1.8 µm, 2.1 mm × 100 mm); solvent system, water (0.04% acetic acid): acetonitrile (0.04% acetic acid); gradient program, 95:5 *v/v* at 0 min, 5:95 *v/v* at 11.0 min, 5:95 *v/v* at 12.0 min, 95:5 *v/v* at 12.1 min, 95:5 *v/v* at 14.0 min; flow rate, 0.35 mL/min; temperature, 40 °C; injection volume: 2 μL. The effluent was alternatively connected to an ESI-triple quadrupole-linear ion trap (Q TRAP)-MS. Linear ion trap (LIT) and triple quadrupole (QQQ) scans were acquired on an ESI 7500 QTRAP LC/MS/MS System, equipped with an ESI turbo ion-spray interface operating in a positive ion mode and controlled by Sciex OS 2.1.6 software (AB Sciex). The operating parameters of the ESI source were as follows: ion source, turbo spray; source temperature, 500 °C; ion-spray (IS) voltage, 2500 V; ion source gas I (GSI), gas II (GSII), and curtain gas (CUR) set at 45, 75, and 45.0 psi, respectively; and collision gas (CAD) set at 11 psi. Instrument tuning and mass calibration were performed with 10 and 100 µmol/L polypropylene glycol solutions in the QQQ and LIT modes, respectively. QQQ scans were acquired in multiple reaction monitoring (MRM) mode, and the collision energy (CE) for individual MRM transitions was determined and further optimized. A specific set of MRM transitions was monitored for each stage in accordance with the metabolites eluting during that stage.

After obtaining the metabolite spectrum analysis data of different samples, we used MultiQuant 3.0.3 to perform peak area integration on the mass spectrum peaks of all metabolites to obtain the content of the metabolites in these samples. And analyzed the mass spectrum peaks according to the metabolite retention time and peak shape information to ensure the accuracy of the quantification.

### 5.7. Metabolome Data Analysis

The filtered data were submitted to Simca-P software (version 13.0, Umetrics AB, Umea, Sweden) for unsupervised PCA and OPLS-DA. Hierarchical clustering analysis of the metabolites between the samples was performed using R software (R version 4.0. www.r-project.org (accessed on 1 October 2021)). For identifying DAMs, a fold change ≥ 2 or a fold change ≤ 0.5 was used as the screening criteria.

## Data Availability

No new data were created or analyzed in this study. Data sharing is not applicable to this article.

## Supplementary Materials

The following are available online at https://www.mdpi.com/article/10.3390/metabo12080691/s1: Figure S1. The graphical abstract of this article as follows; Figure S2. Differences in ester content in coconut flesh between two varieties (HT and GD) over three growth stages. Student’s *t* test was performed (ns: not significant, ** *p* < 0.01, *** *p* < 0.001); Figure S3. PCA score plot of LC-MS metabolomes between two varieties of coconut over three growth stages; Figure S4. Differences in amino acid relative content between two coconut varieties over three growth stages. Student’s *t* test was performed (ns: not significant); Figure S5. Differences in vitamin relative content between two coconut varieties over three growth stages. Student’s *t* test was performed (ns: not significant, * *p* < 0.05, ** *p* < 0.01, *** *p* < 0.001); Table S1. Characterization of volatiles from two coconut varieties over three growth stages by GC-MS; Table S2. Quantitative results of volatiles from two coconut varieties over three growth stages by GC-MS; Table S3. Statistics of differentially accumulating volatiles from two coconut varieties over three growth stages; Table S4. Characterization of compounds from two coconut varieties over three growth stages by LC-MS; Table S5. Quantitative results of metabolites from two coconut varieties over three growth stages by LC-MS; Table S6. Statistics of differentially accumulating metabolite from two coconut varieties over three growth stages.
