# Supplementary material for: Comparative Metabolomics Reveals Key Determinants in the Flavor and Nutritional Value of Coconut by HS-SPME/GC-MS and UHPLC-MS/MS
Source: Metabolites. 2022 Jul 26;12(8):691. doi: 10.3390/metabo12080691 (PMC9394352; doi:10.3390/metabo12080691)

Comparative metabolomics reveals key determinants in the flavor and nutritional value of coconut by HS-SPME/GC-MS and UHPLC-MS/MS

Metabolites

Hao Guo [1,†], Jun Lai [1,†], Chun Li [1,†], Haihong Zhou [1], Chao Wang [1], Weizhen Ye [1], Yue Zhong [1], Xuecheng Zhao [2], Feng Zhang [3], Jun Yang [1,2,*] and Shouchuang Wang [1,2,*]

1 College of Tropical Crops, Hainan University, Haikou 570228, China; haoguo@hainanu.edu.cn (H.G.); jun.lai@hainanu.edu.cn (J.L.); chun.li@hainanu.edu.cn (C.L.); haihong.zhou@hainanu.edu.cn (H.Z.); chaowang@hainanu.edu.cn (C.W.); weizhen.ye@hainanu.edu.cn (W.Y.); yue.zhong@hainanu.edu.cn (Y.Z.);

2 Sanya Nanfan Research Institute of Hainan University, Hainan Yazhou Bay Seed Laboratory, Sanya 572025, China; xczhao@hainanu.edu.cn (X.Z.);

3 National Key Laboratory of Crop Genetic Improvement and National Center of Plant Gene Research (Wuhan), Huazhong Agricultural University, Wuhan 430070, China; zhangfeng@mail.hzau.edu.cn (F.Z.);

* Correspondence: yang9yj@hainanu.edu.cn (J. Y.); shouchuang.wang@hainanu.edu.cn (S. W.)

† These authors contributed equally to this work.

Figure S1. Overview of the flowchart of this study.

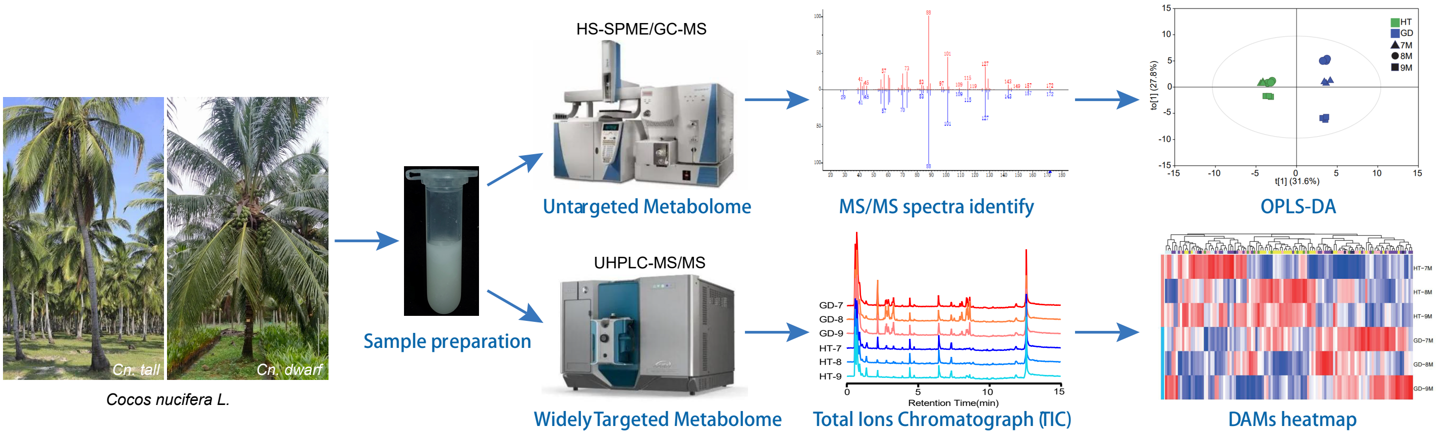

Figure S2. Differences in ester content in coconut flesh between two varieties (HT and GD) over three growth stages. Student's t test was performed (ns: not significant, ** $P< 0.01$, *** $P< 0.001$).

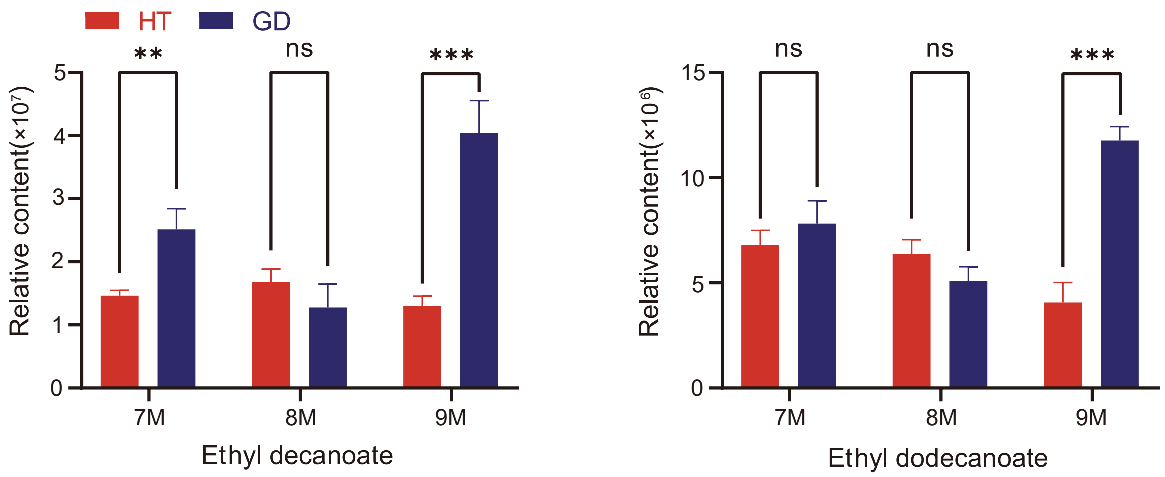

Figure S3. PCA score plot of LC-MS metabolomes between two varieties of coconut over three growth stages.

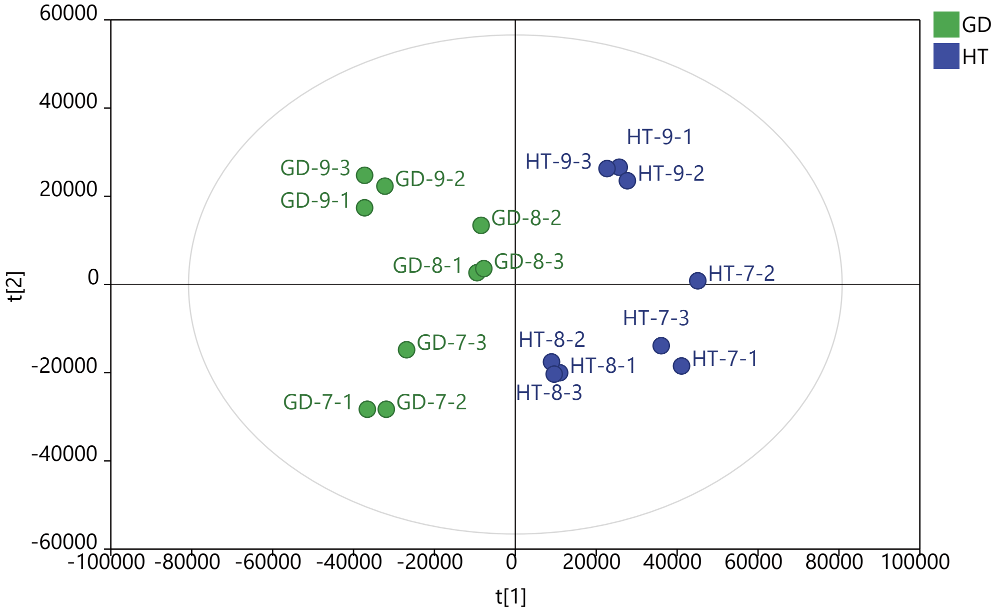

Figure S4. Differences in amino acid relative content between two coconut varieties over three growth stages. Student's *t* test was performed (ns: not significant).

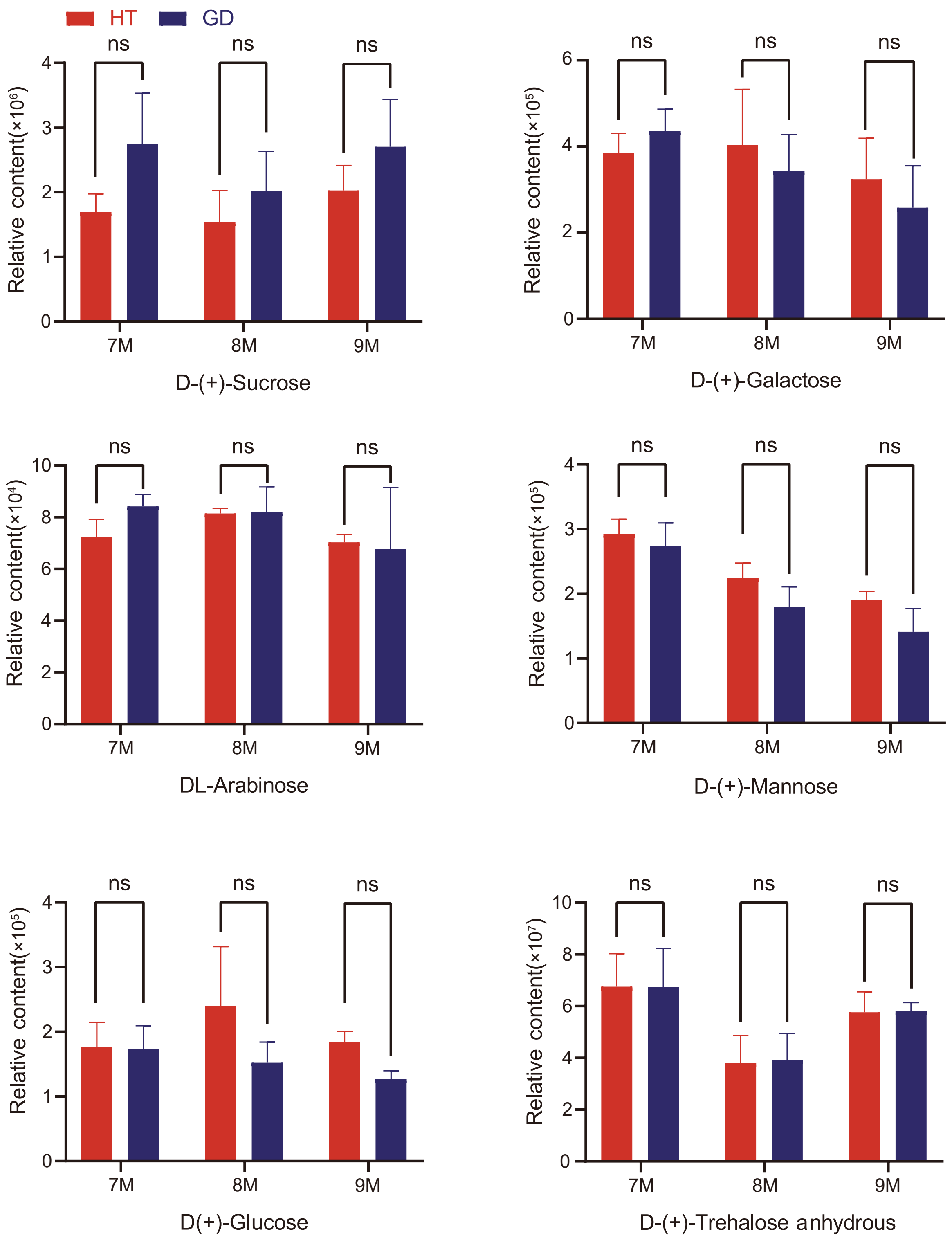

Figure S5. Differences in vitamin relative content between two coconut varieties over three growth stages. Student's *t* test was performed (ns: not significant, * $P < 0.05$, ** $P < 0.01$, *** $P < 0.001$).

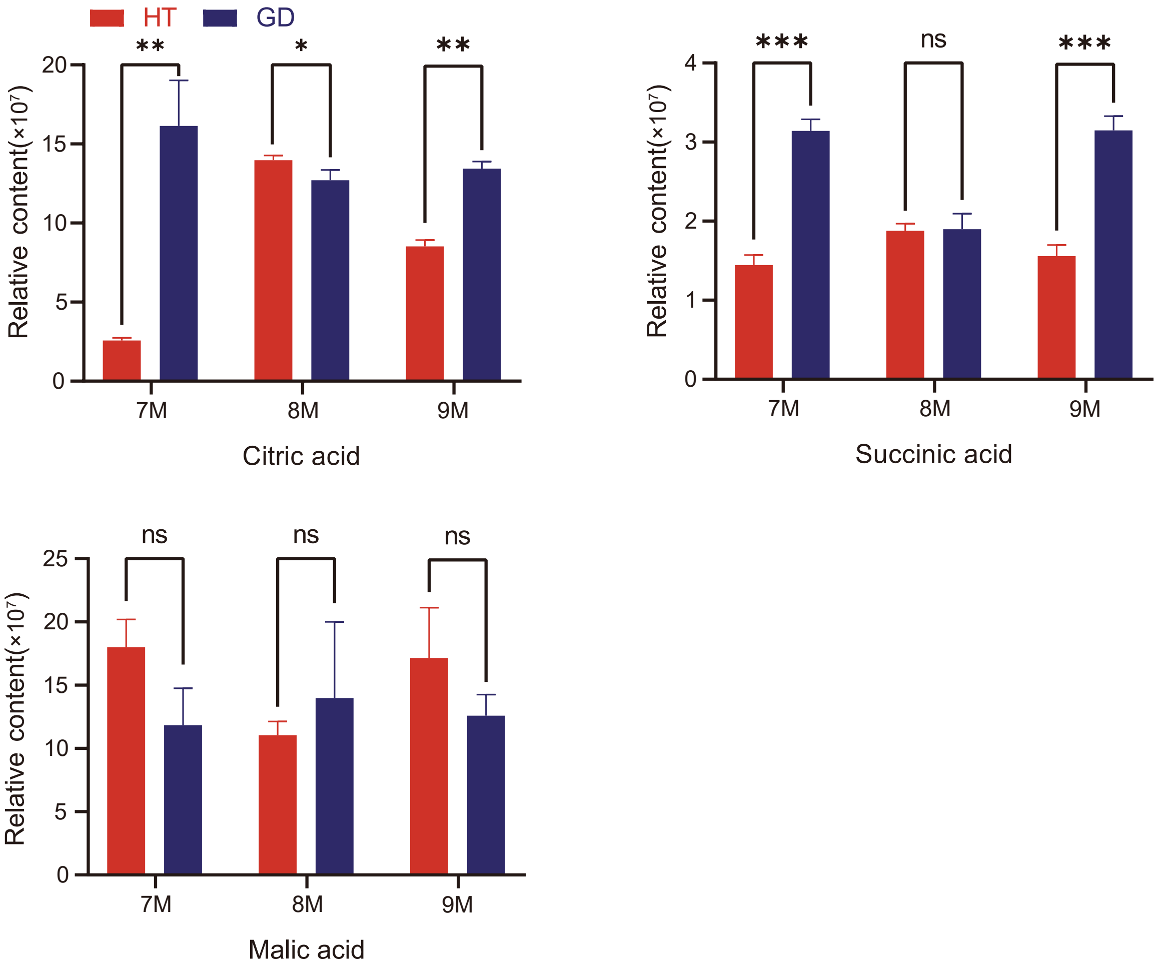

Supplement: Supplementary file 1 [file metabolites-12-00691-s001.zip › Supplementary Figures.pdf]
